# The Maternal Omega-3 Long-Chain Polyunsaturated Fatty Acid Concentration in Early Pregnancy and Infant Neurodevelopment: The ECLIPSES Study

**DOI:** 10.3390/nu16050687

**Published:** 2024-02-28

**Authors:** Behnaz Shahabi, Carmen Hernández-Martínez, Núria Voltas, Josefa Canals, Victoria Arija

**Affiliations:** 1 Research Group in Nutrition and Mental Health (NUTRISAM), Universitat Rovira i Virgili, 43201 Reus, Spain; behnaz.shahabi@urv.cat (B.S.); carmen.hernandez@urv.cat (C.H.-M.); nuria.voltas@urv.cat (N.V.); josefa.canals@urv.cat (J.C.); 2 Pere Virgili Institute for Health Research (IISPV), Universitat Rovira i Virgili, 43201 Reus, Spain; 3 Research Center for Behavioral Assessment (CRAMC), Universitat Rovira i Virgili, 43003 Tarragona, Spain; 4 Serra Húnter Fellow, Department of Psychology, Faculty of Education Sciences and Psychology, Universitat Rovira i Virgili, 43007 Tarragona, Spain

**Keywords:** serum concentrations, omega-3, DHA, EPA, infant neurodevelopment, cognitive development, pregnancy

## Abstract

Omega-3 Long-Chain Polyunsaturated Fatty Acids (*n*-3 LCPUFAs) play a key role in early neurodevelopment, but evidence from observational and clinical studies remains inconsistent. This study investigates the association between maternal *n*-3 LCPUFA, Docosahexaenoic Acid (DHA), and eicosapentaenoic acid (EPA) concentrations during pregnancy and infant development functioning at 40 days. This study includes 348 mother–infant pairs. Maternal serum concentrations were assessed in the first and third trimesters alongside sociodemographic, clinical, nutritional, psychological, and obstetrical data. At 40 days, the Bayley Scales of Infant and Toddler Development, Third Edition (BSID-III) was administered. An adjusted analysis revealed that lower first-trimester *n*-3 LCPUFA and DHA concentrations are associated with better infant motor development. These results underscore the potential significance of the maternal *n*-3 LCPUFA status in early pregnancy for influencing fetal neurodevelopment. However, the complexity of these associations necessitates further investigation, emphasizing the urgent need for additional studies to comprehensively elucidate the nuanced interplay between the maternal *n*-3 LCPUFA status and infant neurodevelopment.

## 1. Introduction

The first 1000 days of life have been recognized as a critical period for the optimal growth and development of an individual. During this period, nutrition is the non-genetic factor with the most influence [1]. The developing brain is highly susceptible to nutritional influences, and adequate nutrient status during pregnancy is vital for promoting optimal cognitive and neurological outcomes in offspring [2]. Within this context, *n*-3 LCPUFAs (including alpha-linolenic acid (ALA), DHA, and EPA) play a vital role in early neurodevelopment, as they are crucial for the formation and functioning of neuronal membranes, synapses, and neurotransmitter release and for regulating overall brain function and development [3,4,5]. To carry out their function, *n*-3 LCPUFA and, concretely, DHA, are accumulated in human tissues during gestation and the first two years of life [6,7,8], which is a period of rapid brain development in humans [9]. Therefore, in humans, while it is widely recognized that the maternal prenatal intake of essential *n*-3 LCPUFA is crucial for the optimal development and functioning of the central nervous system (CNS) in infants [10], studies present conflicting results. Most existing evidence regarding the association between *n*-3 LCPUFA and children’s neurodevelopment stems from Randomized Controlled Trial (RCT) studies of maternal supplementation during pregnancy. While some studies conducted in developed countries found that DHA supplementation during pregnancy [11,12,13] did not have an effect on infant cognitive development in 9- and 18-month-old and 5-year-old infants, others observed a positive effect [14]. In this sense, Cochrane reviews and meta-analyses of these RCTs have shown that the results are inconclusive and that there is insufficient evidence to support that *n*-3 LCPUFA supplementation during gestation has a positive impact on neurodevelopment [15,16,17,18,19]. Another source of evidence comes from observational studies about the maternal prenatal intake of DHA-rich foods, which have shown associations with better infant development [20,21,22,23,24,25,26,27].

Studies about maternal prenatal concentrations are limited in number and have yielded less conclusive findings. Rioux et al. [28] assessed the maternal prenatal DHA status in the third trimester of pregnancy, and assessed infant cognitive development at six months using the Bayley Scales of Infant Development (BSID) in a cohort of 63 mother–infant pairs, and found that there is no significant association between DHA and infant performance. In older Dutch children, Brouwer-Brolsma et al. [29] examined the maternal concentrations of arachidonic acid (AA), DHA, and EPA in plasma phospholipids in the third trimester of pregnancy and child cognitive function at seven years without finding significant associations. However, there are additional studies that have established links between *n*-3 LCPUFA and infant cognitive performance. For example, a study conducted in Seychelles investigated the connections between maternal PUFAs, methylmercury, and infant cognitive development at 9 and 30 months of age [30]. The results demonstrated a positive correlation between psychomotor development in 9-month-old infants and total *n*-3 LCPUFA levels during the third trimester. Similarly, a study conducted on 12-month-old Norwegian children found that maternal DHA concentrations in the third trimester were associated positively with infants’ problem-solving abilities [31]. As can be observed, few studies have assessed maternal *n*-3 LCPUFA concentrations during the whole gestation, and the results are inconclusive and difficult to compare due to variations in *n*-3 LCPUFA measurements across different tissues and the assessment of cognitive abilities at different ages when other significant factors may have influences.

Most of the studies presented (observational and RCT), which investigate the impact of several *n*-3 LCPUFAs on child neurodevelopment, have been carried out mainly in older infants and children, and they hardly provide data on the maternal serum levels of fatty acids resulting from supplementation or observation. Given the complexity of early fetal brain development, it is essential to understand which *n*-3 LCPUFA serum levels are related to neurodevelopment and at which stage of pregnancy, including early pregnancy, and to expand this knowledge to cover very young infants. Thus, the main aim of this study is to analyze the association of the maternal *n*-3 LCPUFA concentration during pregnancy with early neurodevelopment in infants, adjusting the relationship for other related factors in a population of women from the Mediterranean region of Spain.

## 2. Materials and Methods

### 2.1. Study Design and Procedure

**Figure 1:**
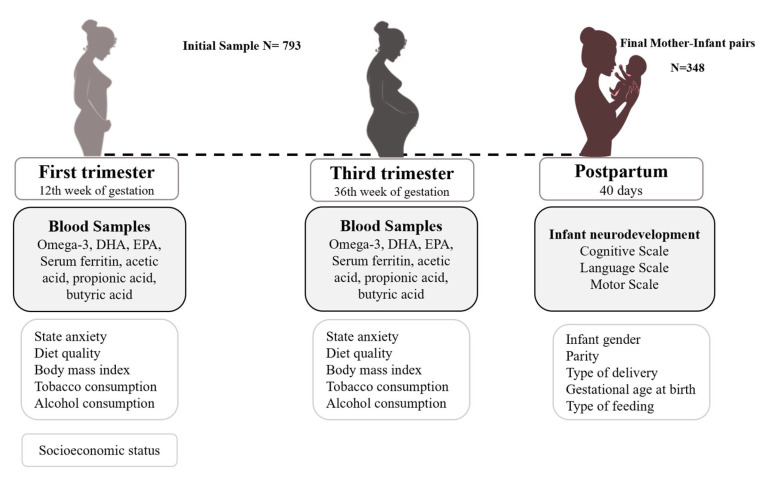
Flow chart of the study design, measurements, and population.

This is a prospective follow-up study of pregnant women from the first trimester to 40 days postpartum. This study is part of the ECLIPSES data research project in Tarragona, Catalonia, Spain [32,33]. Before the 12th week of pregnancy, individuals were chosen from primary care institutions, and they were closely monitored at weeks 12, 24, and 36 of pregnancy, as well as on the 40th day after giving birth. Eligible participants were at least 18 years old, within their first 12 weeks of pregnancy, and free from anemia (Hb > 110 g/L). Multiple pregnancies, previous severe diseases such as immunosuppression, or any chronic disease that can affect nutritional development (malabsorption syndrome, diabetes, cancer, or hepatopathies) were the exclusion criteria in this study. Samples of blood and data related to sociodemographic, clinical, and psychosocial factors were gathered. Serum concentrations of *n*-3 LCPUFA, DHA, and EPA were determined in the 12th and 36th weeks of gestation in a random subsample of 450 participants. Out of the initial 450 participants, 348 took part in the postpartum evaluation, during which their infants were examined. As a result, this study’s ultimate sample size was 348 mother–infant pairs. A flow chart of participants and measurements is provided in Figure 1.

The Clinical Research Ethics Committee of the Jordi Gol Institute for Primary Care Research (IDIAP), the Pere Virgili Institute for Health Research (IISPV) (REF.NO. 118/2017, date: 2017), and the Spanish Agency for Medicines and Medical Devices (AEMPS) approved all procedures involving human subjects. Every woman who took part in the study gave her informed permission. Also, this clinical trial was registered at www.clinicaltrialsregister.eu, accessed on 21 May 2013 with EudraCT number 2012-005480-28, and at www.clinicaltrials.gov, accessed on 23 June 2017 with identification number NCT03196882. All participants signed an informed consent form. The study complies with the tenets of the Declaration of Helsinki.

### 2.2. Instruments and Data Collection

#### 2.2.1. Main Measurements 

Blood samples were obtained from pregnant women who were fasting. After centrifugation, the serum was kept cold—at −80 °C—until further examination. LC-MS/MS was used to measure serum concentrations of *n*-3 LCPUFA, DHA, and EPA. Briefly, a standard internal mixture was mixed with 20 μL of serum in methanol to precipitate proteins. The supernatant was mixed with water, O-Benzylhydroxylamine (BHA, Sigma-Aldrich, St. Louis, MO, USA), and *N*-(3 Dimethylaminopropyl)-*N*0-ethyl carbodiimide (EDC, Sigma-Aldrich) to obtain *n*-3 LCPUFA derivatives. *n*-3 LCPUFA derivatives were purified by liquid–liquid extraction using diethyl ether and by ultra-performance liquid chromatography-mass spectrometry (UHLC-MS/MS) using a UHPLC 1290 Infinity II Series coupled to a QqQ 6470 Series^®^ (Agilent Technologies Inc., Santa Clara, CA, USA) and analyzed. Chromatographic separation was performed by gradient elution using a ternary mobile phase containing water, methanol, and isopropanol with ammonium formate on a Kinetex Polar C18 analytical column (2.6 μm, 2.1 × 100 mm) (Phenomenex, Torrance, CA, USA). The mass spectrometer was operated in multiple reaction monitoring (MRM) modes, and PUFAs were ionized by positive electrospray. The UHPLC-MS/MS system was monitored by an Agilent MassHunter^®^ workstation (Agilent Technologies Inc., Santa Clara, CA, USA). 

Infants’ neurodevelopment was assessed using the BSID-III [34], an individually administered assessment designed to assess the current developmental status of language and motor skills and four specific subscales (expressive language, receptive language, fine motor, and gross motor). At 40 days old, the cognitive scale focused on assessing infants’ sensorimotor development, visual attention, exploration, self-regulation ability, and habituation to environmental infants aged 0 to 42 months. The BSID-III comprises three general scales (cognitive, stimuli, and recognition of the primary caregiver). The receptive language subscale examined an infant’s capacity for pre-verbal communication skills, sound differentiation, attention to and perception of environmental stimuli, as well as orientation towards social and environmental stimuli. Meanwhile, the expressive language subscale assessed pre-verbal communication, encompassing behaviors such as smiles, early vocalizations, and babbling. The motor scale included the fine motor subscale, evaluating an infant’s hand position, visual object tracking, and response to tactile stimuli, and the gross motor subscale appraised an infant’s muscular tone, limb and torso movement, static positioning, and cephalic control. Two trained psychologists conducted the BSID-III assessment at the 40-day postpartum visit in the presence of both parents.

#### 2.2.2. Adjustment Measurements

##### Prenatal Psychological Distress

The State-Trait Anxiety Inventory (STAI) in Spanish was used to assess the symptoms of mother anxiety [35], which includes 40 items that assess state anxiety (transient and situational anxiety level) and trait anxiety (dispositional and stable trait anxiety level). In this study, the state anxiety score in the first and third trimesters was used. 

##### Sociodemographic Data

The women’s socioeconomic statuses (SESs) were calculated using the Hollingshead index [36] by merging data about the mothers’ and couples’ (if the mothers had a partner) levels of education and profession, classified according to the Catalan classification of occupations (CCO-2011), to obtain a total score representing the family SES [37].

##### Lifestyle Habits

The diet quality scores of pregnant women were estimated in the first and third trimesters according to their adherence to the Mediterranean diet and by using a Food Intake Frequency Questionnaire (FFQ) [38,39]. The process used to obtain the scores was as follows: The FFQ, which contains 45 food and beverage items, was completed according to the amount of each item consumed per week or month. Based on the daily gram of consumption for each substance, nine groups were formed: fruits (seeds, nuts, and fruits, but not fruit juices), legumes, vegetables, cereals (wholemeal and refined flour, rice, pasta, other grains, and bread), fresh fish (fish and seafood), all meat (fresh meat and processed meat), olive oil, dairy products (milk, cheese, creamy desserts, and yogurt), and alcoholic beverages. Each group was stated in grams per 1000 kcal/day, and tertiles were obtained to assign a categorical score related to low, medium, and high intakes (0, 1, and 2, respectively). To calculate the total rMED score, the nine scores were added together, taking into account that six groups scored positively (fruit, legumes, vegetables, cereals, olive oil, and fresh fish) and three components scored negatively (total meat, dairy products, and alcohol); thus, the total rMED score ranged from 0 points (minimum adherence to the Mediterranean diet) to 18 points (maximum adherence to the Mediterranean diet). The FFQs were carried out by qualified midwives and nutritionists who monitored the reviewing and entering of the food data and the data scrubbing and analysis.

Smoking during pregnancy was assessed using the Fagerström questionnaire (Fagerström_Q) [40], and women were divided into two groups: smokers and non-smokers.

Alcohol use during pregnancy was evaluated based on the mothers’ responses to the alcoholic beverages item in the FFQ questionnaire, and it was categorized as either “yes” or “no” depending on the response.

##### Clinical Data

The mothers’ anthropometric measures were weight (kg) and height (cm), and Body Mass Index (BMI) was calculated from these measures (weight (kg)/height (m^2^)). BMI was computed at the first and third trimesters, and BMI change from the first to the third trimester was determined.

The number of alive sons (parity) was obtained from clinical records.

Fasting serum samples were collected throughout the first and final trimesters of pregnancy. Serum ferritin levels were measured through immune chemiluminescence. 

Serum short-chain fatty acids (SCFAs) (acetic acid, propionic acid, and butyric acid) were quantified by liquid chromatography– mass spectrometry (LC-MS/MS). Then, the supernatants were mixed with water, o-Benzylhydroxylamine (BHA, Sigma Aldrich, St. Louis, MO, USA), and *N*-(3-dimethylaminopropyl)-*N*′-ethylcarbodiimide (EDC, Sigma Aldrich) to obtain amount of SCFAs derived from AGCC. SCFA derivatives were purified by liquid–liquid extraction using diethyl ether, and SCFA quantification was performed via LC-MS/MS using the Series 1290 Infinity II Ultra High-Performance Liquid Chromatography (UHPLC) coupled to a QqQ 6470 Series^®^ (Agilent Technologies Inc., Santa Clara, CA, USA). Chromatographic separation was performed with gradient elution using a ternary mobile phase containing water, methanol, and isopropanol with ammonium formate on the Kinetex Polar C18 analytical column (2.6 μm 2.1 × 100 mm) (Phenomenex, Torrance, CA, USA). The mass spectrometer operates in multiple reaction monitoring (MRM) mode, and SCFAs were ionized by positive electrospray. The UHPLC-MS/MS system was controlled by the Agilent MassHunter^®^ workstation (Agilent Technologies Inc., Santa Clara, CA, USA). Samples were analyzed in duplicate, and the average of the two values was calculated.

##### Obstetrical and Birth Data

Gestational age at birth and type of delivery were collected from the babies’ health cards. The mothers were also asked about the type of feeding they used. The genders of the babies were also recorded.

### 2.3. Statistical Analysis

The description of the community was expressed using descriptive statistics, in which the data are expressed as mean and standard deviation (SD) for quantitative variables and number of cases and percentage (%) for qualitative variables. 

Tertiles of *n*-3 LCPUFA, DHA, and EPA serum concentrations at the first and third trimesters were computed to obtain groups at each trimester. After that, ANCOVA analyses were performed to assess the differences in infant cognitive development scores at 40 days old according to maternal *n*-3 LCPUFA, DHA, and EPA tertiles in the first and third trimesters. The adjustment variables used were the following: family socioeconomic status (low/mid/high); mother’s state anxiety (total score) in the first/third trimesters; tobacco consumption (yes/no); alcohol consumption (yes/no); BMI change from first to third trimester (kg/m^2^); quality of diet in the first/third trimesters (total score); serum ferritin (μg/L) in the first/third trimesters; SCFAs (acetic acid, propionic acid, and butyric acid) in the first/third trimesters; parity (nulliparous/multiparous); gestational age at birth (weeks); infant’s gender (boy/girl); type of delivery (eutocic/dystocic); and type of feeding (formula/breastfeeding). We calculated the estimated adjusted means and utilized the Bonferroni post hoc analysis to assess significant differences between the *n*-3 LCPUFA, DHA, and EPA level groups.

To test the predictive and continuous association between *n*-3 LCPUFA and DHA in the first trimester and the 40-day-old infant neurodevelopment scores, multiple linear regression models using the enter method were performed. The variables entered in the model to adjust the relationship were the same as the ANCOVA adjustment variables.

The significance level was set at 0.05. All the statistical analyses were run by the statistical software package SPSS version 29.0 for Windows (SPSS, Chicago, IL, USA).

## 3. Results

### 3.1. General Characteristics of the Sample

The characteristics of the participants are shown in Table 1. Overall, the mean age of the pregnant women was 38.8 years (SD = 5.1), and approximately 60% had a middle to high socioeconomic status.

### 3.2. Infant Cognitive Development According to Mothers’ n-3 LCPUFA, DHA, and EPA Levels in First and Third Trimesters

Infant cognitive development mean scores and estimated mean scores after adjusting for covariables according to mothers’ *n*-3 LCPUFA, DHA, and EPA tertile groups in first and third trimesters are shown in Table 2, Table 3, and Table 4, respectively.

Regarding *n*-3 LCPUFA, the infants of mothers in tertile 1 in the first trimester showed significantly higher scores in the motor index (adjusted mean = 110.165) and the gross motor index (adjusted mean = 11.679) than the infants of mothers in tertile 3 (adjusted mean = 105.736 and 10.744, respectively). In the third trimester, no significant relationship was observed (Table 2).

Similar results were found regarding DHA (Table 3). The infants of mothers in tertile 1 showed significantly higher scores in motor index (adjusted mean = 109.710) and gross motor index (adjusted mean = 11.669) than the infants of mothers in tertile 3 (adjusted mean = 105.208 and 10.595, respectively). 

Concerning the EPA, Table 4 shows no significant difference.

### 3.3. Predictive Relationship between n-3 LCPUFA and DHA Serum Levels at First Trimester and Infant Motor and Gross Motor Development

The findings of the multivariate-adjusted linear regression models examining the associations between the maternal levels of *n*-3 LCPUFA and DHA in the first trimester and infant cognitive development are shown in Table 5. Both the maternal *n*-3 LCPUFA and DHA concentrations in the first trimester showed significant negative relationships with the motor index scores (β = −0.015, *p* = 0.034 and β = −0.021, *p* = 0.029, respectively) and gross motor scores (β = −0.004, *p* = 0.012 and β = −0.005, *p* = 0.003, respectively). The infants’ motor index and gross motor scores slightly decreased with greater maternal *n*-3 LCPUFA and DHA levels during the first trimester of pregnancy. 

## 4. Discussion

This study assessed the maternal serum concentrations of circulating *n*-3 LCPUFA, DHA, and EPA during the first and third trimesters of pregnancy in a large sample of healthy women from a Mediterranean region of Spain. We investigated the impact of these concentrations on the neurodevelopment of their infants at forty days of age. Our findings indicate that lower maternal levels of *n*-3 LCPUFA and DHA during the first trimester are related to better motor scores in infants, particularly in gross motor skills, while there is no relationship between EPA and infant neurodevelopment scores.

To date, there are no established reference ranges for LCPUFA concentrations. Nevertheless, the *n*-3 LCPUFA concentrations observed in our sample resembles those reported in other samples of healthy pregnant women from diverse countries, including the Netherlands, Hungary, Finland, England, and Ecuador [41], as well as those documented in the Human Metabolome Database [42]. Considering this, the results of our study introduce a paradox by questioning the conventional belief that high maternal levels of *n*-3 LCPUFA can improve infant neurodevelopment. However, findings from specific studies examining maternal concentrations during pregnancy and infant neurodevelopment also differ from this widely accepted idea. To our knowledge, the Maastricht Essential Fatty Acid Cohort (MEFAB) study is the only one that has specifically examined associations between maternal concentrations of AA, DHA, and EPA in plasma phospholipids in each trimester of pregnancy and child cognitive and academic performances at the age of 7 [29,43]. Their results showed that there was no significant relationship with school cognitive performance and a negative association between prenatal DHA in the first trimester and child arithmetic skills, indicating that higher levels were detrimental to the development of these areas. The study concluded that the negative associations observed call for prudence when considering DHA supplementation during pregnancy [43]. Regarding studies assessing *n*-3 LCPUFA concentrations in the third trimester and at the end of pregnancy, mixed findings have been reported. While some studies reported improved psychomotor development in 9-month-old infants, higher problem-solving abilities in 12-month-old infants, and better language skills in 5-year-old children of mothers with higher concentrations of *n*-3 LCPUFA and DHA [30,31,44], others did not find significant associations with cognitive performance in 6-month-old babies [28]. Additionally, there has not been a reported association between EPA and infant neurodevelopment. These findings highlight the complexity of interpreting the relationship between maternal *n*-3 LCPUFA concentrations and infant neurodevelopment, given that crucial factors influencing this association remain inadequately studied. Such factors include the timing of pregnancy examined, the methods employed for assessing infant neurodevelopment, and the lack of comprehensive data on the PUFA serum concentrations in most studies, including variations in measurement methods and expressions (% of total fatty acids or serum concentrations), as well as the assessment of the confounding factors of the relationship.

The age of the child and the methodology employed for neurodevelopmental assessment can significantly contribute to variability in the results. In this context, the motor development domain assessed by the BSID-III, which is used in this study, assesses indicators of muscular tone and maturity, encompassing limb and torso movement, static positioning, and cephalic control—all linked to the maturity of the CNS [45,46]. Thus, our results suggest that infants born to mothers in the low tertile of *n*-3 LCPUFA and DHA during the first trimester may exhibit a more mature CNS.

Given the limited existing evidence and the intricate interpretation of the association between maternal fatty acid concentrations and child neurodevelopment, it is noteworthy to consider the extensive research conducted in supplementation studies during pregnancy, with a primary focus on DHA supplementation or dietary supplements like fish oil. These studies indirectly allow us to observe whether an increase in the serum levels of fatty acids has a positive effect on infant neurodevelopment. However, these studies have also yielded contradictory results. On the one hand, recent reviews suggest that beneficial effects are discernible primarily in specific subgroups, such as preterm infants or pregnant women with low *n*-3 LCPUFA levels, genetic predisposition, or unfavorable socioeconomic and educational characteristics [10,47,48,49]. However, in many cases, high-dose DHA supplementation had null or even adverse effects on children’s neurodevelopment [10]. The authors of a recent review [10] indicate that these adverse effects have been poorly mentioned in previous reviews and meta-analyses, probably because it is difficult to discern the mechanisms by which DHA supplementation may have adverse effects. Although intervention studies of supplementation do not provide information on maternal serum levels or the specific trimester in which the effects on infant neurodevelopment are occurring, they reported the harm caused by high doses of *n*-3 LCPUFA, which supports our findings.

Concerning our results and the evidence presented, we can hypothesize that in the first trimester, when the embryo is small in size, lower amounts of *n*-3 LCPUFA may adequately meet the fetal requirements for proper development, while higher doses may be detrimental, probably due to oxidation and inflammation processes.

Once fetal fatty acid needs are met, excess fatty acids can undergo various metabolic pathways that contribute to the production of Reactive Oxygen Species (ROS) that overwhelm the cellular antioxidant and immune systems, leading to an imbalance known as oxidative stress [50]. This oxidative stress can result in cellular damage, including lipid peroxidation, protein oxidation, and DNA damage, ultimately affecting cellular function [51]. In the context of neurodevelopment, oxidative balance is fundamental to all cellular and neuronal processes, including neurogenesis, synaptogenesis, neuronal migration, and pruning [52]. Disruptions to this balance can have detrimental effects on the CNS, leading to neurodevelopmental dysfunctions [53]. Animal studies have demonstrated the detrimental oxidative effects of high levels of fatty acids, with overfed mice receiving DHA showing decreased lifespans, likely due to oxidative stress and reduced cellular function [54]. Nevertheless, further human studies are needed to confirm these suggestions.

The development of neurological circuits during the embryonic and fetal phases is a highly complex process influenced by multiple genetic, biological, metabolic, and environmental factors, among which maternal diet during pregnancy, nutritional status, the presence of viral infections, stress, medication use, and even the maternal microbiota stand out [55]. In our study, we have adjusted the multivariate analysis for several of these factors, such as sociodemographic characteristics (family socioeconomic status and parity); psychological distress (mother’s anxiety symptoms); clinical aspects (body mass index during pregnancy); nutritional factors (mother’s serum ferritin levels and quality of diet during pregnancy); lifestyle habits (tobacco and alcohol use); obstetric outcomes (gestational age at birth, type of delivery, and infant’s gender); mother’s acetic, propionic, and butyric acid levels (as indicators of mother microbiota); and infant breastfeeding at the moment of cognitive assessment. This adjustment has allowed us to isolate the effect of maternal *n*-3 LCPUFA concentrations on fetal neurodevelopment from the rest of the mentioned adjusting factors.

Various strengths of our study have been mentioned previously, such as the assessment of the *n*-3 LCPUFA concentration in the serum at the beginning and end of pregnancy, as well as the exhaustive control of confounders in our analysis. However, the limitations of our study need to be contextualized to interpret our results with caution. Of the group of omega-3 fatty acids, α-linolenic acid (ALA) could not be incorporated in this work because only trace levels were detected. We assessed infant neurodevelopment using the BSID-III. Although it is a widely recognized and internationally used tool specifically designed and validated for assessing cognitive development from birth to 42 months, it is crucial to acknowledge the inherent limitations of early childhood neurodevelopment assessment, which tends to exhibit notable variability [56,57]. Recognizing this inherent variability is essential for interpreting our results cautiously. Nevertheless, it is important to note that in our previous studies, we have identified associations between various maternal prenatal nutritional states and other environmental exposures and neurodevelopment assessed at this stage using the same tool [58,59]. This highlights the complexity and multifactorial nature of early neurodevelopment, underscoring the need for careful consideration when interpreting our findings in the broader context of infant development. On the other hand, the inclusion of a substantial number of participants enhances the generalizability of our findings to similar populations but limits the generalizability to other populations with different dietary patterns and genetic backgrounds. Therefore, we posit that these findings may be applicable to Mediterranean populations in southern Europe sharing similar sociocultural and environmental characteristics. Consequently, the replication of our study across diverse populations would help validate the observed associations and explore potential variations. Moreover, further research is needed to explore the long-term implications of maternal fatty acid levels on cognitive and neurological performance in children. Overall, these findings contribute to our understanding and can guide professionals in supporting optimal prenatal nutrition and neurodevelopmental outcomes.

## 5. Conclusions

In conclusion, our study findings indicate that, in a community sample of well-nourished, healthy pregnant women from a Mediterranean area, lower serum levels of *n*-3 LCPUFA and DHA in the first trimester of pregnancy are associated with improved neurodevelopmental scores in infants 40 days after birth. The levels observed in this sample may be adequate during early embryonic stages, while the higher levels observed might potentially induce adverse effects by fostering oxidation and inflammation processes, which could negate the beneficial effects. Although these results seem to partially support the evidence from some previous studies, it is crucial to acknowledge that further studies are needed to confirm and expand upon these findings. The replication of our study in larger and more diverse populations, as well as longitudinal studies assessing long-term neurodevelopmental outcomes, would provide a stronger evidence base. 

## Figures and Tables

**Table 1:** Descriptive data of the mothers and offspring: sociodemographic, lifestyle, nutrition, and psychological data (*n* = 348).

Maternal Characteristics during Pregnancy	
Age (years), mean ± SD	38.8 ± 5.1
Family socioeconomic level, *n* (%)	
Low	144 (41.4)
Mid	152 (43.7)
High	52 (14.9)
Mother’s anxiety (total score) during pregnancy, mean ± SD	17.5 ± 8.8
Quality of diet in the first/third trimesters (total score), mean ± SD	9.9 ± 2.1
Tobacco consumption, *n* (%)	
No	296 (85.1)
Yes	52 (14.9)
Alcohol consumption, *n* (%)	
No	298 (85.5)
Yes	50 (14.5)
BMI change in the first and third trimesters (kg/m^2^), mean ± SD	4.0 ± 1.4
Serum ferritin (µg/L), mean ± SD	
First trimester	40.9 ± 28.3
Third trimester	16.2 ± 9.1
Acetic acid (μmol/L), mean ± SD	
First trimester	49.4 ± 21.6
Third trimester	48.6 ± 18.5
Propionic acid (μmol/L), mean ± SD	
First trimester	3.5 ± 0.9
Third trimester	3.5 ± 1.0
Butyric acid (μmol/L), mean ± SD	
First trimester	0.7 ± 0.3
Third trimester	0.8 ± 0.4
*n*-3 LCPUFA (μmol/L), mean ± SD	
First trimester	281.2 ± 96.9
Tertile 1	180.7 ± 28.3
Tertile 2	264.8 ± 23.7
Tertile 3	390.8 ± 65.6
Third trimester	261.5 ± 83.1
Tertile 1	175.3 ± 23.8
Tertile 2	247.4 ± 22.5
Tertile 3	359.4 ± 52.6
DHA (μmol/L), mean ± SD	
First trimester	242.5 ± 75.0
Tertile 1	162.4 ± 24.5
Tertile 2	233.6 ± 21.1
Tertile 3	326.3 ± 46.2
Third trimester	237.0 ± 70.5
Tertile 1	164.1 ± 21.8
Tertile 2	224.6 ± 18.6
Tertile 3	319.0 ± 43.6
EPA (μmol/L), mean ± SD	
First trimester	37.5 ± 25.6
Tertile 1	14.0 ± 4.0
Tertile 2	29.8 ± 5.1
Tertile 3	65.3 ± 23.2
Third trimester	24.0 ± 16.7
Tertile 1	8.5 ± 3.4
Tertile 2	19.1 ± 3.6
Tertile 3	42.9 ± 13.9
**Obstetrical outcomes**	
Previous parity, *n* (%)	
Nulliparous	191 (55.0)
Multiparous	157 (45.0)
Gestational age at birth (weeks), mean ± SD	39.8 ± 1.2
Type of delivery, *n* (%)	
Eutocic	228(65.6)
Dystocic	120 (34.4)
**Baby characteristics**	
Infant’s gender, *n* (%)	
Boy	185 (53.2)
Girl	163 (46.8)
Type of feeding, *n* (%)	
Formula	64 (18.4)
Breastfeeding	284 (81.6)
**Infant cognitive development, mean ± SD**	
Cognitive scale	102.10 ± 8.0
Language scale	96.5 ± 8.2
Receptive	10.6 ± 2.0
Expressive	8.1 ± 1.5
Motor scale	107.7 ± 11.5
Fine	11.5 ± 1.9
Gross	11.0 ± 2.2

Values are expressed as mean ± SD (standard deviation) or *n* = number (%). Abbreviations: DHA, docosahexaenoic acid; EPA, eicosapentaenoic acid; BMI, body mass index.

**Table 2:** *n*-3 LCPUFA levels at the first and third trimesters of pregnancy and neurodevelopmental offspring data.

*n*-3 LCPUFA		First Trimester					Third Trimester				
		Tertile 1 ^a^ < 224.3653 μmol/L	Tertile 2 ^b^ 224.3653 μmol/L–312.6929 μmol/L	Tertile 3 ^c^ > 312.692 μmol/L	*p*	Post Hoc	Tertile 1 ^a^ < 211.6078 μmol/L	Tertile 2 ^b^ 20,211.6078 μmol/L–287.8421 μmol/L	Tertile 3 ^c^ > 287.8421 μmol/L	*p*	Post Hoc
Cognitive Index	Unadjusted mean (SD)	102.87 (8.0)	102.30 (8.8)	100.95 (7.4)	0.190		102.64 (7.6)	101.10 (9.3)	102.59 (7.3)	0.267	
	Adjusted mean	102.572	102.523	100.952	0.255		102.888	101.059	102.133	0.265	
Language Index	Unadjusted mean (SD)	96.69 (7.4)	96.91 (8.9)	96.12 (8.4)	0.758		97.09 (7.7)	95.48 (9.0)	97.13 (8.1)	0.234	
	Adjusted mean	96.476	97.013	96.103	0.725		97.369	95.114	96.590	0.149	
Receptive	Unadjusted mean (SD)	10.77 (1.9)	10.72 (2.2)	10.48 (2.2)	0.553		10.81 (1.8)	10.33 (2.3)	10.94 (2.0)	0.070	
	Adjusted mean	10.616	10.739	10.520	0.755		10.833	10.282	10.846	0.101	
Expressive	Unadjusted mean (SD)	8.07 (1.5)	8.19 (1.6)	8.17 (1.6)	0.843		8.18 (1.5)	8.09 (1.7)	8.05 (1.6)	0.847	
	Adjusted mean	8.150	8.196	8.134	0.956		8.240	8.015	7.966	0.437	
Motor Index	Unadjusted mean (SD)	**110.23 (10.3)**	**107.35 (10.9)**	**105.58 (13.2)**	**0.011**	**a–c 0.009**	108.23 (9.8)	107.20 (11.0)	107.64 (13.8)	0.804	
	Adjusted mean	**110.165**	**106.603**	**105.736**	**0.025**	**a–c 0.028**	108.534	106.630	107.075	0.509	
Fine	Unadjusted mean (SD)	11.67 (1.9)	11.44 (1.9)	11.38 (1.9)	0.508		11.55 (2.0)	11.46 (2.0)	11.58 (1.8)	0.883	
	Adjusted mean	11.669	11.321	11.391	0.438		11.613	11.321	11.530	0.554	
Gross	Unadjusted mean (SD)	**11.72 (2.3)**	**10.95 (2.3)**	**10.71 (2.2)**	**0.003**	**a–b 0.035** **a–c 0.003**	11.10 (2.0)	10.97 (2.4)	11.18 (2.4)	0.794	
	Adjusted mean	**11.679**	**10.863**	**10.744**	**0.010**	**a–b 0.038** **a–c 0.014**	11.112	10.929	11.079	0.840	

a: Tertile 1 group; b: Tertile 2 group, c: Tertile 3 group. Statistical models adjusted using the ANCOVA for family socioeconomic status (low/mid/high); mother’s anxiety (total score) during pregnancy; tobacco consumption (yes/no); alcohol consumption (yes/no); BMI change from the first to the third trimester (kg/m^2^); quality of diet in the first/third trimesters (total score); serum ferritin (μg/L) in the first/third trimesters; acetic acid (μmol/L) in the first and third trimesters; propionic acid (μmol/L) in the first and third trimesters; butyric acid (μmol/L) in the first and third trimesters; parity (nulliparous/multiparous); gestational age at birth (weeks); infant’s gender (boy/girl); type of delivery (eutocic/dystocic); type of feeding (formula/breastfeeding). The significance of the numbers is shown in bold. Abbreviation: BMI, body mass index. The *p* values obtained are Bonferroni-corrected, and those <0.05 are considered significant and are represented in bold.

**Table 3:** Docosahexaenoic acid levels at the first and third trimesters of pregnancy and neurodevelopmental offspring data.

Docosahexaenoic Acid		First Trimester					Third Trimester				
		Tertile 1 ^a^ < 199.0497 μmol/L	Tertile 2 ^b^ 199.0497 μmol/L –268.8105 μmol/L	Tertile 3 ^c^ > 268.8105 μmol/L	*p*	Post Hoc	Tertile 1 ^a^ < 195.1726 μmol/L	Tertile 2 ^b^ 195.1726 μmol/L –264.4524 μmol/L	Tertile 3 ^c^ > 264.4524 μmol/L	*p*	Post Hoc
**Cognitive Index**	Unadjusted mean (SD)	102.73 (8.0)	102.48 (8.8)	100.90 (7.5)	0.182		102.05 (8.1)	101.40 (9.1)	102.83 (7.0)	0.417	
	Adjusted mean	102.444	102.667	100.907	0.228		102.358	101.233	102.442	0.477	
**Language Index**	Unadjusted mean (SD)	96.75 (7.6)	96.55 (9.1)	96.72 (7.8)	0.982		96.58 (7.7)	95.70 (9.2)	97.31 (8.0)	0.344	
	Adjusted mean	96.346	96.744	96.796	0.919		96.954	95.107	96.896	0.193	
**Receptive**	Unadjusted mean (SD)	10.68 (1.9)	10.67 (2.2)	10.68 (2.1)	0.999		**10.71 (1.9)**	**10.30 (2.3)**	**11.03 (2.0)**	**0.029**	**b–c 0.024**
	Adjusted mean	10.495	10.705	10.727	0.710		10.787	10.174	10.963	0.057	
**Expressive**	Unadjusted mean (SD)	8.17 (1.5)	8.12 (1.6)	8.19 (1.5)	0.937		8.10 (1.4)	8.19 (1.8)	8.03 (1.6)	0.732	
	Adjusted mean	8.215	8.144	8.169	0.948		8.149	8.110	7.959	0.657	
**Motor Index**	Unadjusted mean (SD)	**110.08 (10.7)**	**107.87 (10.5)**	**105.10 (13.1)**	**0.005**	**a–c 0.004**	108.49 (9.9)	106.47 (11.0)	108.11 (13.6)	0.386	
	Adjusted mean	**109.710**	**107.448**	**105.208**	**0.029**	**a–c 0.024**	108.688	105.907	107.596	0.252	
**Fine**	Unadjusted mean (SD)	11.59 (2.0)	11.55 (1.9)	11.38 (1.9)	0.671		11.50 (2.0)	11.41 (2.0)	11.66 (1.8)	0.612	
	Adjusted mean	11.548	11.493	11.365	0.800		11.557	11.272	11.616	0.399	
**Gross**	Unadjusted mean (SD)	**11.75 (2.3)**	**11.01 (2.2)**	**10.55 (2.2)**	**<0.001**	**a–b 0.044** **a–c >0.001**	11.24 (2.0)	10.78 (2.4)	11.24 (2.4)	0.219	
	Adjusted mean	**11.669**	**10.949**	**10.595**	**0.004**	**a–c 0.003**	11.222	10.759	11.138	0.321	

a: Tertile 1 group; b: Tertile 2 group, c: Tertile 3 group. Statistical models adjusted using the ANCOVA for family socioeconomic status (low/mid/high); mother’s anxiety (total score) during pregnancy; tobacco consumption (yes/no); alcohol consumption (yes/no); BMI change from first to the third trimester (kg/m^2^); quality of diet in the first/third trimesters (total score); serum ferritin (μg/L) in the first/third trimesters; acetic acid (μmol/L) in the first and third trimesters; propionic acid (μmol/L) in the first and third trimesters; butyric acid (μmol/L) in the first and third trimesters; parity (nulliparous/multiparous); gestational age at birth (weeks); infant’s gender (boy/girl); type of delivery (eutocic/dystocic); type of feeding (formula/breastfeeding). The significance of the numbers is in bold. Abbreviation: BMI, body mass index. The *p* values obtained are Bonferroni-corrected, and those <0.05 are considered significant and are represented in bold.

**Table 4:** Eicosapentaenoic acid levels at the first and third trimesters of pregnancy and neurodevelopmental offspring data.

Eicosapentaenoic Acid		First Trimester				Third Trimester			
		Tertile 1 ^a^ < 20.9746 μmol/L	Tertile 2 ^b^ 20.9746 μmol/L –38.6014 μmol/L	Tertile 3 ^c^ > 38.6014 μmol/L	*p*	Tertile 1 ^a^ < 14.2267 μmol/L	Tertile 2 ^b^ 14.2267 μmol/L –27.1558 μmol/L	Tertile 3 ^c^ > 27.1558 μmol/L	*p*
**Cognitive Index**	Unadjusted mean (SD)	102.78 (7.8)	102.03 (8.5)	101.59 (7.5)	0.556	102.36 (8.2)	102.41 (8.2)	101.52 (8.0)	0.655
	Adjusted mean	102.925	102.166	101.287	0.373	102.590	102.190	101.300	0.500
**Language Index**	Unadjusted mean (SD)	96.38 (7.6)	97.29 (8.5)	96.22 (8.4)	0.572	96.72 (8.2)	96.14 (8.3)	96.97 (8.7)	0.741
	Adjusted mean	96.266	97.479	96.049	0.400	96.810	95.769	96.611	0.645
**Receptive**	Unadjusted mean (SD)	10.77 (2.0)	10.81 (2.2)	10.47 (2.1)	0.395	10.73 (2.0)	10.62 (2.2)	10.76 (2.1)	0.873
	Adjusted mean	10.639	10.866	10.451	0.366	10.751	10.515	10.737	0.687
**Expressive**	Unadjusted mean (SD)	7.97 (1.5)	8.23 (1.6)	8.22 (1.5)	0.398	8.13 (1.5)	8.03 (1.6)	8.18 (1.6)	0.755
	Adjusted mean	8.055	8.241	8.171	0.714	8.135	8.004	8.079	0.850
**Motor Index**	Unadjusted mean (SD)	108.49 (10.4)	108.09 (11.2)	106.72 (13.1)	0.499	108.07 (10.4)	107.50 (10.3)	107.42 (14.0)	0.906
	Adjusted mean	108.463	107.844	106.318	0.444	108.106	107.345	106.736	0.726
**Fine**	Unadjusted mean (SD)	11.37 (1.8)	11.48 (2.0)	11.64 (1.9)	0.599	11.42 (2.0)	11.53 (1.9)	11.58 (1.9)	0.828
	Adjusted mean	11.380	11.433	11.568	0.799	11.446	11.469	11.490	0.988
**Gross**	Unadjusted mean (SD)	11.41 (2.4)	11.18 (2.3)	10.83 (2.2)	0.168	11.17 (2.2)	10.96 (2.3)	11.14 (2.3)	0.749
	Adjusted mean	11.356	11.160	10.799	0.249	11.143	10.954	11.054	0.853

a: Tertile 1 group; b: Tertile 2 group, c: Tertile 3 group. Statistical models adjusted using the ANCOVA for family socioeconomic status (low/mid/high); mother’s anxiety (total score) during pregnancy; tobacco consumption (yes/no); alcohol consumption (yes/no); BMI change from first to third trimester (kg/m^2^); quality of diet in the first/third trimesters (total score); serum ferritin (μg/L) in the first/third trimesters; acetic acid (μmol/L) in the first and third trimesters; propionic acid (μmol/L) in the first and third trimesters; butyric acid (μmol/L) in the first and third trimesters; parity (nulliparous/multiparous); gestational age at birth (weeks); infant’s gender (boy/girl); type of delivery (eutocic/dystocic); type of feeding (formula/breastfeeding). Abbreviation: BMI, body mass index.

**Table 5:** Multivariate-adjusted linear regression models for the associations of maternal *n*-3 LCPUFA, and DHA levels and infants’ neurodevelopment in the first trimester.

	Motor Index		Gross Motor			Motor Index		Gross Motor	
	β	*p*	β	*p*		β	*p*	β	*p*
*n*-3 LCPUFA (μmol/L)	−0.015	**0.034**	−0.004	**0.012**	DHA (μmol/L)	−0.021	**0.029**	−0.005	**0.003**
SES (low/mid/high)	−0.010	0.854	0.013	0.232	SES (low/mid/high)	−0.011	0.847	0.012	0.260
Mother’s anxiety (total score)	−0.011	0.886	0.006	0.710	Mother’s anxiety (total score)	−0.012	0.873	0.005	0.728
Tobacco consumption (Yes/No)	4.453	**0.022**	0.849	**0.024**	Tobacco consumption (Yes/No)	4.349	**0.025**	0.817	**0.031**
Alcohol consumption (Yes/No)	1.845	0.324	0.316	0.384	Alcohol consumption (Yes/No)	1.771	0.342	0.318	0.379
BMI change (kg/m^2^)	0.098	0.839	−0.032	0.730	BMI change (kg/m^2^)	0.100	0.834	−0.037	0.695
Quality of diet (total score)	0.335	0.275	0.040	0.507	Quality of diet (total score)	0.326	0.282	0.031	0.593
Serum ferritin (μg/L)	0.008	0.742	0.005	0.277	Serum ferritin (μg/L)	0.010	0.691	0.005	0.248
Acetic acid (μmol/L)	0.080	**0.032**	0.011	0.140	Acetic acid (μmol/L)	0.080	**0.033**	0.010	0.169
Propionic acid (μmol/L)	−1.703	0.077	−0.344	0.067	Propionic acid (μmol/L)	−1.651	0.087	−0.325	0.082
Butyric acid (μmol/L)	−1.441	0.559	0.183	0.703	Butyric acid (μmol/L)	−1.457	0.553	0.221	0.643
Parity (nulliparous/multiparous)	2.540	0.077	0.388	0.164	Parity (nulliparous/multiparous)	2.528	0.077	0.410	0.140
Gestational age at birth (weeks)	1.403	**0.011**	0.225	**0.036**	Gestational age at birth (weeks)	1.368	**0.012**	0.234	**0.027**
Infant’s gender (boy/girl)	0.459	0.734	−0.032	0.902	Infant’s gender (boy/girl)	0.430	0.749	−0.061	0.817
Type of delivery (eutocic/dystocic)	0.607	0.680	0.079	0.783	Type of delivery (eutocic/dystocic)	0.619	0.672	0.072	0.799
Type of infant feeding (formula/breastfeeding)	0.961	0.584	0.367	0.283	Type of infant feeding (formula/breastfeeding)	0.944	0.590	0.352	0.301
Adjusted global model	R^2^ × 100 = 4.4 F_16/294_ = 1.895 *p* = 0.021		R^2^ × 100 = 4.7 F_16/294_ = 1.961 *p* = 0.015			R^2^ × 100 = 4.5 F_16/295_ = 1.923 *p* = 0.018		R^2^ × 100 = 5.8 F_16/295_ = 2.195 *p* = 0.005	
Adjusted model with significant variables	R^2^ × 100 = 4.7 F_4/329_ = 5.138 *p* = 0.001		R^2^ × 100 = 4.5 F_3/333_ = 6.221 *p* = 0.000			R^2^ × 100 = 4.9 F_4/330_ = 5.328 *p* = 0.000		R^2^ × 100 = 5.5 F_3/334_ = 7.598 *p* = 0.000	

The significance of the numbers in bold is *p*-value < 0.05.

## Data Availability

The datasets generated and/or analyzed during the study are not publicly accessible due to considerations of subject confidentiality. However, they can be obtained from the corresponding author upon reasonable request.
